# Prevalence of macrosomic newborn and maternal and neonatal complications in a high-risk maternity

**DOI:** 10.61622/rbgo/2024rbgo48

**Published:** 2024-06-27

**Authors:** Kellen Silva Sousa, Henrique Vitor Leite, Mário Dias Corrêa, Matheus Silva Sousa, Anna Luíza Rocha Queiroz

**Affiliations:** 1 Universidade Federal de Minas Gerais Belo Horizonte MG Brasil Universidade Federal de Minas Gerais, Belo Horizonte, MG, Brasil.

**Keywords:** Fetal macrosomia, Fetal weight, Diabetes, gestational, Shoulder dystocia, Jaundice, neonatal, Pregnancy, high risk, Risk factors

## Abstract

**Objective:**

Evaluate the prevalence of macrosomic newborns (birth weight above 4000 grams) in a high-risk maternity from 2014 to 2019, as well as the maternal characteristics involved, risk factors, mode of delivery and associated outcomes, comparing newborns weighing 4000-4500 grams and those weighing above 4500 grams.

**Methods:**

This is an observational study, case-control type, carried out by searching for data in hospital’s own system and clinical records. The criteria for inclusion in the study were all patients monitored at the service who had newborns with birth weight equal than or greater than 4000 grams in the period from January 2014 to December 2019, being subsequently divided into two subgroups (newborns with 4000 to 4500 grams and newborns above 4500 grams). After being collected, the variables were transcribed into a database, arranged in frequency tables. For treatment and statistical analysis of the data, *Excel* and *R* software were used. This tool was used to create graphs and tables that helped in the interpretation of the results. The statistical analysis of the variables collected included both simple descriptive analyzes as well as inferential statistics, with univariate, bivariate and multivariate analysis.

**Results:**

From 2014 to 2019, 3.3% of deliveries were macrosomic newborns. The average gestational age in the birth was 39.4 weeks. The most common mode of delivery (65%) was cesarean section. Diabetes mellitus was present in 30% of the deliveries studied and glycemic control was absent in most patients. Among the vaginal deliveries, only 6% were instrumented and there was shoulder dystocia in 21% of the cases. The majority (62%) of newborns had some complication, with jaundice (35%) being the most common.

**Conclusion:**

Birth weight above 4000 grams had a statistically significant impact on the occurrence of neonatal complications, such as hypoglycemia, respiratory distress and 5th minute APGAR less than 7, especially if birth weight was above 4500 grams. Gestational age was also shown to be statistically significant associated with neonatal complications, the lower, the greater the risk. Thus, macrosomia is strongly linked to complications, especially neonatal complications.

## Introduction

Macrosomia can be defined as a birth weight equal to or greater than 4000 grams, regardless of gestational age, in the most of studies, or even as a weight equal to or greater than 4500 grams in a smaller amount of them.^(1)^

According to the Technical Manual of High-Risk Pregnancy of the Ministry of Health and the American College of Obstetricians and Gynecologists (ACOG), the cutoff point for this diagnosis is 4000 grams, emphasizing that the risk of morbidity for newborns with this weight is higher than the general obstetric population and increases markedly when birth weight is greater than 4500 grams.^(2-4)^

Data extracted from the Department of Informatics of the Unified Health System (DATASUS) show that in 2020, of the total of 2,730,145 live births in Brazil, 147,649 (5.4%) had a registered weight of 4000 grams or more.^(5)^ In the United States, in 2020, according to data from the National Center for Health Statistics (NCHS), the prevalence is slightly higher: 8% of newborns were born with more than 4000 grams.^(6)^

The main maternal risk factors related to macrosomia listed in the literature are diagnosis of diabetes mellitus, history of a macrosomic newborn in a previous pregnancy, obesity, and maternal weight gain. Regarding diabetes mellitus, a major work conducted, the HAPO (Hyperglycemia and Adverse Pregnancy Outcomes) Study Cooperative Research Group, which sought to find the adverse outcomes associated with hyperglycemia in pregnancy, indicated strong association between maternal glucose levels and birth weight of the newborn.^(7)^ In addition to being a risk factor for the occurrence of macrosomia alone, diabetes mellitus has been associated with different anthropometric measurements and body composition of newborns, compared to macrosomic newborn measurements of non-diabetic mothers, which may explain the increased risk of shoulder dystocia in this population.^(8)^ Thus, control of maternal hyperglycemia reduces the risk of macrosomia, and its adequate management is highly recommended.^(3,9)^

Maternal complications are often related to cephalopelvic disproportion (CPD), and include prolonged labor, shoulder dystocia, uterine rupture, cesarean delivery, puerperal hemorrhage, and 3rd and 4th degree perineal lacerations.^(10,11)^ Studies show that the risk of cesarean section for women who attempt a vaginal delivery of newborns weighing more than 4500 grams is at least twice that of the control group, with the indication for cesarean section being attributed to dystocia in the labor.^(4)^ Neonatal complications of macrosomia can also be serious, including brachial plexus and skeletal injuries, secondary to shoulder dystocia, meconium aspiration syndrome, perinatal asphyxia, hypoglycemia, jaundice and even death.^(10)^

Prenatal prediction of macrosomia is often inaccurate. A systematic review evaluated the accuracy of two-dimensional (2D) ultrasonographic biometry for predicting macrosomia found that it was an overall poor predictor, regardless of estimated fetal weight.^(12-15)^

The timing of the termination of pregnancy and the decision on the mode of delivery can often be conflicting when macrosomia is suspected. With limited or inconsistent scientific evidence, ACOG recommends (Level B) that suspected fetal macrosomia should not be an indication for induction of labor before 39 weeks. As Level C evidence, scheduled cesarean delivery could be beneficial for newborns who have an estimated fetal weight of at least 5000 grams in women without diabetes and an estimated fetal weight of at least 4500 grams in women with diabetes.^(3)^

In view of the scientific relevance of the proposed subject, the main objective of this study is to evaluate the prevalence of macrosomic newborns in a high-risk maternity hospital from 2014 to 2019, as well as the maternal characteristics involved, the factors of risk, mode of delivery and associated maternal and neonatal complications, comparing newborns weighing between 4000-4500 grams and those weighing more than 4500 grams.

## Methods

This is an observational study, case-control type, conducted from the search for data, in own system (SISMATER) and clinical records, of deliveries that occurred at Maternity Hospital of the Hospital das Clínicas of UFMG (Federal University of Minas Gerais).

The criteria for inclusion in the study were all patients monitored at the service who had newborns with birth weight equal than or greater than 4000 grams in the period from January 2014 to December 2019, being subsequently divided into two subgroups (newborns with 4000 to 4500 grams and newborns above 4500 grams). No distinctions were made regarding race, social group or associated maternal diseases for inclusion in the project. Fetuses with malformations and stillbirths were not excluded from the sample.

The primary variable analyzed were women who had a newborn with birth weight equal or greater than 4000 grams (the criteria for inclusion in the study). Once this subgroup was selected, the following variables were evaluated: age, multiparity or nulliparity, gestational age at delivery, mode of delivery and its indication, Robson classification, estimated fetal weight at ultrasound (if the last ultrasound was performed within 15 days before delivery), presence or absence of maternal diabetes mellitus (diagnosed prior to pregnancy or not, according Pan American Health Organization,^(16)^control of diabetes mellitus (with pre and post-prandial glycemic profile), maternal history of macrosomic newborn or previous cesarean delivery due to cephalopelvic disproportion.

After this initial data collection, these patients were classified into two subgroups (newborns with 4000 to 4500 grams and newborns above 4500 grams) for the further outcomes analysis: occurrence of shoulder dystocia, need for instrumented delivery, presence of puerperal hemorrhage (requiring the use of Misoprostol and/or blood transfusion to assess the severity of the hemorrhage), perineal lacerations (especially 3rd and 4th degrees), episiotomy, occurrence of birth trauma, AGPAR of 5 minutes less than 7, neonatal ICU admission, neonatal complications (jaundice, respiratory distress, hypoglycemia). Neonatal hypoglycemia was defined behind capilar glycemic measure. The measure was realized in all newborns with birth weight above 4000 grams in: 2, 4 and 8 hours of life. If diabetics mother’s newborn: 1, 2, 4, 8, 12 and 24 hours. Hypoglycemia was defined when glycemic capilar below 45 in the first 24 hours.

After being collected, the variables were transcribed into a database, arranged in frequency tables. For treatment and statistical analysis of the data, *Excel* and *R* software were used.^(13)^ This tool was used to create graphs and tables that helped in the interpretation of the results.

The total number of rows in the Database (367) represents the number of deliveries with occurrence of macrosomia in the studied period, and, knowing the total number of deliveries, it was possible to have an unbiased estimate of the prevalence of macrosomia. Allied to this punctual estimate, a Confidence Interval was calculated for such percentage π, following the score method.^(14)^

In the univariate analysis, the categorical (or qualitative) variables were presented through their respective frequency tables (absolute and proportional). A Confidence Interval with 95% confidence (α=0.05) was calculated for the percentages of each answer and each variable using the score method. Quantitative variables were presented using the following descriptive statistics: mean, standard deviation, median, minimum, and maximum. A Confidence Interval was constructed for the calculated means, if the Central Limit Theorem applies here, and that the sample means have a normal distribution.^(15)^

In the bivariate analysis, the analysis between the independent variables and responses was done in two ways, depending on whether the variables were categorical or quantitative. Independent variables selected for the bivariate analysis were:

1. Categorical variables: “Multiparous”, “Diabetes mellitus”, “Weight > p 90 in the last ultrasound”, “Weight ≥ 4000 grams in the last ultrasound”, “History of macrosomia”, “Birth weight > 4500 grams”.
2. Quantitatives variables: “Gestational age”, “Number of gestations” e “Birth weight”.

The outcomes (responses variables), in which we compared the groups (newborns with 4000 to 4500 grams and newborns above 4500 grams), were: “Shoulder Dystocia”, “Birth trauma”, “Instrumented delivery”, “Neonatal Complications”, “Neonatal Jaundice”, “Neonatal hypoglycemia”, “Neonatal respiratory distress”, “Newborns admitted to the Neonatal ICU”, “5-minute APGAR score of less than 7”, “3rd or 4th degree lacerations”, “Episiotomy”, “Increased puerperal bleeding”.

For the categorical independent variables, cross tables were created, the Odds Ratios were calculated, followed by the p-values according to the Chi-Square test, using the *chisq.test* function of *R*. Confidence intervals for the Odds Ratios were calculated using the normal approximation of the distribution of the natural logarithm of the Odds Ratio estimate, also known as the Wald method. And in the case where OR = 0, the upper limit of the Interval was calculated using a correction.^(14)^ For the quantitative independent variables, the means, medians, and standard deviations were calculated for each outcome of the response variable, and such medians were compared using the parametric Student’s t test, using the *t.test* function of *R*.^(15)^

In cases where the results of the bivariate analysis described above showed more than one variable statistically associated with an outcome (p-value<0.05), they were compared together in a multivariate analysis using logistic regression, through the *glm* function of *R*.^(14)^

This study was conducted in accordance with the Declaration of Helsinki (2000) and following the guidelines and standards contained in Resolution CNS 466/12. The information of interest was obtained exclusively for research purposes. The principle of secrecy was preserved by keeping the patients’ personal data anonymous. The dissemination of research results is done without mentioning the name or any form of identification of the participants. Because it is a database query, the present study waived the need to sign an informed consent form. The study was approved by the Research Ethics Committee (COEP) 3.536.293 of the Federal University of Minas Gerais and has no conflict of interest. CAAE: 12038118.1.0000.5149.

## Results

The ratio of macrosomic newborns and the total number of births in the period from 2014 to 2019 at the Maternity Hospital of the Hospital das Clínicas of UFMG show a prevalence of 3.3% of macrosomia (Table 1). Of these, 49 newborns weighed 4500 grams or more, which represents 13.3% of the total number of macrosomic newborns.

**Table 1:** Total number of births and number of macrosomic newborns at Maternity Hospital of the Hospital das Clínicas of UFMG from 2014 to 2019

Year	Total deliveries	Number of deliveries with macrosomic newborns	Prevalence (%)
2014	1,578	64	4.1
2015	1,991	70	3.5
2016	1,851	54	2.9
2017	1,978	65	3.3
2018	1,851	70	3.8
2019	1,732	44	2.5
Total	10,981	367	3.3

Regarding the number of previous births, most women (77%) were multiparous and, 15% of pregnant women already had a history of previous children weighing more than 4000 grams (Table 2).

**Table 2:** Maternal characteristics of pregnant women who had macrosomic newborns (n=367)

Variables	Average	IC 95% Average	Standard deviation	Median	Minimum	Maximum	n
Patient’s age	28.6	(27.9 – 29.2)	6.2	28	14	43	367
Gestational age	39.4	(39.3 – 39.6)	1.4	40	29	42	366
Number of pregnancies	2.4	(2.3 – 2.6)	1.3	2	1	7	367
Number of normal births	1.1	(1 – 1.2)	1.3	1	0	6	367
Number of cesarean deliveries	1.1	(1 – 1.2)	1.0	1	0	5	367
Number of abortions	0.3	(0.2 – 0.3)	0.6	0	0	3	367

Diabetes mellitus was present in 30% of the women, and of this total, 13% already had diabetes when they became pregnant, and 59% were diagnosed with Gestational Diabetes Mellitus (GDM) during the prenatal according to the criteria used at the Hospital das Clínicas in the analyzed period (at least two fasting blood glucose measurements between 92 and 125 were necessary to obtain the diagnosis of GDM in the first trimester of pregnancy). Another 28% were not diagnosed during the prenatal follow-up/hospitalization, but would be diagnosed by the current criteria suggested by the Ministry of Health (just one blood glucose measurement greater than 92 is sufficient to validate such a diagnosis).^(16)^ In this group, the diagnosis was made retrospectively during data collection for this study (the information of GDM’s diagnosis wasn’t contained upon medical register and the authors needed to check the patient’s exams and disease’s criteria to confirm diagnosis). Control of diabetes mellitus was absent in 62% of patients (Table 3).

**Table 3:** Diagnosis of maternal diabetes mellitus and glycemic control status of pregnant women who had macrosomic newborns

Variables	n (%)	IC%
Diabetes mellitus		
No	256(70)	(65.1 - 74.5)
Yes	111(30)	(25.5 - 34.9)
Moment of diagnosis (diabetes mellitus)		
GDM - Prenatal diagnosis	46(41)	(32.7 - 50.7)
GDM - Diagnosis in childbirth hospitalization	20(18)	(12 - 26.2)
GDM - Retrospective diagnosis	31(28)	(20.4 - 36.9)
Previous diabetes mellitus	14(13)	(7.7 - 20)
No diabetes	256	
Control of Diabetes mellitus (according pre and post-prandial glycemic profile)		
Uncontrolled (with or without medication)	69(62)	(52.9 - 70.7)
Controlled with diet	23(21)	(14.2 - 29.2)
Controlled with insulin	18(16)	(10.5 - 24.2)
Controlled with Metformin	1(0.9)	(0.2 - 4.9)

**Figure 1:**
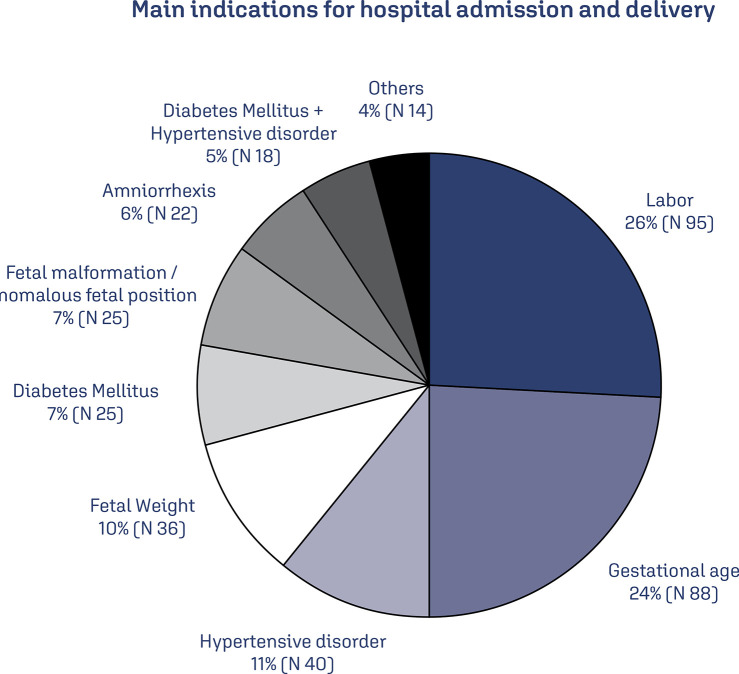
Main indications for hospital admission and delivery

Regarding the indication for hospitalization of the patient, it was identified that the most frequent reasons for admission were labor (26%) and advanced gestational age - 41 weeks (24%). Hypertensive disorders were responsible for 16% of births and diabetes mellitus by 12%. In only 10% of the cases, the weight at the last ultrasound (which was performed within 15 days before delivery) was the only reason for the indication of termination of pregnancy (Figure 1).

**Figure 2:**
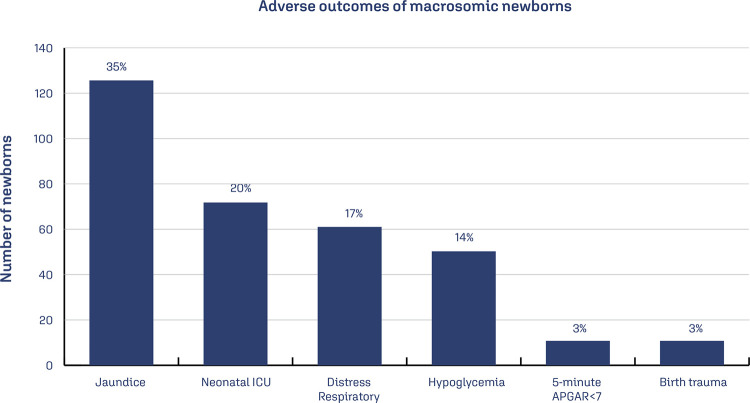
Adverse outcomes of macrosomic newborns

The most common mode of delivery (65%) was cesarean section, and of these, 70% were elective cesarean sections. Among vaginal deliveries, only 6% were instrumented (7 using forceps and 1 vacuum extractor). There was shoulder dystocia in 27 cases (21% of vaginal deliveries). In 22 of them, the dystocia was resolved with the first line of maneuvers - “McRoberts and/or Rubin I”. Regarding neonatal outcomes (Figure 2), most newborns (62%) had some complication, with jaundice (35% of all deliveries) being the most common and birth trauma (which includes brachial plexus injury and/or bone injury) the least common (3% of vaginal deliveries). About 71 newborns (20%) were admitted to the neonatal ICU. Cases of malformation corresponded to 13% (48 cases), with central nervous system’s malformation corresponded to 26 cases. There were 4 stillbirths.

**Figure 3:**
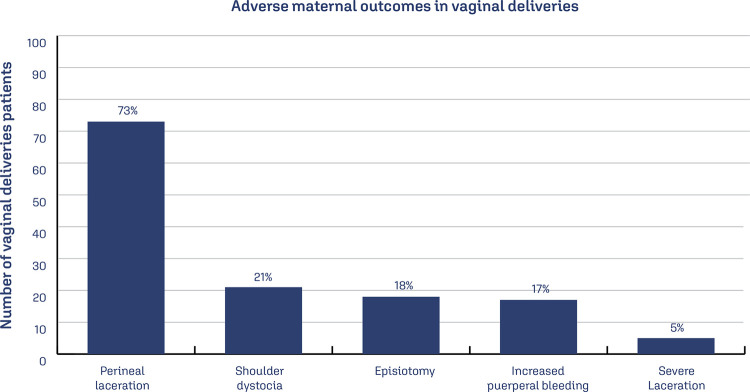
Adverse maternal outcomes

Regarding the outcomes for puerperal women (Figure 3), perineal laceration stood out with 73% of vaginal deliveries. Severe lacerations (3rd or 4th degree) were present in 5% of deliveries. Episiotomy was performed in 24 cases (18%). Increased puerperal bleeding was recorded in 70 patients – 19% (22 in vaginal deliveries and 48 in cesarean deliveries). In 22 cases, rectal administration of Misoprostol was required to control bleeding and in 15 cases (4%) blood transfusion was required.

Bivariate analyzes were performed, and, in some cases, multivariate analyses, organized according to each outcome. Only birth weight above 4500 grams was associated with neonatal complications (p-value 0.005). The risk of having complications was 3 times greater for newborns weighing ≥ 4500 grams than those weighing less than 4500 grams. We can also observe that gestational age and birth weight were associated with neonatal complications. Gestational age had a negative association (p-value 0.000) and weight had a positive association (p-value 0.001). Newborns with complications weighed an average of almost 100 grams more. In this case, it was made a general approach to some possible neonatal complications, like: birth trauma, neonatal jaundice, neonatal hypoglycemia, respiratory distress, low APGAR in the 5th minute and ICU admission. Specific complications are discussed separately. The multivariate analysis presented similar findings, not changing the interpretation of the result. Newborns whose mothers had diabetes mellitus had a 78% higher risk of jaundice than those with mothers without diabetes. It can also be identified that gestational age was associated (negatively) with neonatal jaundice. However, in the multivariate model, neither the diagnosis of diabetes nor the gestational age was statistically significantly associated with the occurrence of neonatal jaundice (p-value 0.087 and 0.066). Newborns weighing more than 4500 grams were 3.27 times more likely to have hypoglycemia than those weighing less than 4500 grams (p-value 0.002). Only “Birth weight”, among the quantitative variables, was associated (positively) with neonatal hypoglycemia (p-value 0.008). Newborns with hypoglycemia weighed an average of 130 grams more than those without hypoglycemia. No categorical variable analyzed was associated with neonatal respiratory distress. Only birth weight was associated (positive association) with this outcome (p-value 0.044). Newborns with respiratory distress had an average weight about 85 grams higher than those without respiratory distress. Newborns admitted to the Neonatal ICU had mean gestational ages lower than those who were not admitted (p-value 0.010). The risk of having a 5-minute APGAR score of less than 7 was about 7 times higher in newborns weighing more than 4500 grams (p-value 0.001). Among the quantitative variables, both “Gestational age” and “Birth weight” were associated with the outcome. In the multivariate model, “Gestational age” is statistically significant (the higher the gestational age, the less chance that the 5th-minute APGAR score is less than 7), with p-value of 0.027, as well as “Birth weight > 4500 grams” (p-value 0.001). No categorical variable was associated with the occurrence of 3rd or 4th degree lacerations. Birth weight was associated (negatively) with the outcome (p-value 0.003). The occurrence of 3rd and 4th degree lacerations was not associated with performing an episiotomy (OR: 0.70 - p-value 1). The risk of episiotomy was higher in nulliparous women compared to multiparous women (OR: 0.16 - p-value 0.000). Multiparous women had a 60% lower risk of increased puerperal bleeding when compared to nulliparous women (p-value 0.003). Finally, it is observed that no quantitative variable was associated with this outcome. The table 4 shows a direct comparative analysis and a resume of the results showed before between newborns weighing between 4000-4500 grams and newborns with birth weight above 4500 grams with regard to the selected outcomes (responses variables).

**Table 4:** A comparison between newborns with birth weight of 4000-4500 grams and newborns above 4500 grams with the responses variables

	Newbons with birth wheight above 4500 grams		
	OR	Confidence Interval	p-value
Shoulder dystocia	2.02	0.58-7.61	0.471
Birth trauma	1.47	0.36-8.23	0.973
Instrumented delivery	0.00	0.00-9.39	0.754
Neonatal complications	3.00	1.35-6.15	0.005
Neonatal jaundice	1.24	0.67-2.34	0.600
Neonatal hypoglycemia	3.27	1.61-6.74	0.002
Neonatal respiratory distress	1.84	0.91-3.86	0.142
Newborns admitted to the Neonatal ICU	1.89	0.96-3.81	0.101
5-minute APGAR score of less than 7	7.26	2.22-23.28	0.001
3rd or 4th degree lacerations	0.00	0.00-7.46	0.626
Episiotomy	0.36	0.06-4.18	0.552
Increased puerperal bleeding	0.44	0.18-1.25	0.133

## Discussion

The prevalence of macrosomia in the Maternity Hospital of the Hospital das Clínicas of UFMG from 2014 to 2019 (3.3%) was slightly lower than the national average. According to data from DATASUS, in the same period, 903,539 newborns weighed more than 4000 grams in Brazil, corresponding to 5.14% of the total number of newborns alive in the period.^(5)^ This difference can be explained by the fact that many of the patients who had their births monitored at the Maternity also had their prenatal care performed at the hospital’s own outpatient clinic. Prenatal care performed at a high-risk reference service allows multidisciplinary follow-up and strict control of risk factors.

A risk factor classically described in the literature for the occurrence of macrosomia is maternal diabetes mellitus, with a prevalence ranging from 1 to 37.7%, with a world average of 16.2%.^(16)^ Although most macrosomics are born to non-diabetic mothers, diabetes remains a well-established risk factor.^(17)^ In our study, 30% of mothers of macrosomic babies were diagnosed with diabetes mellitus (N=111), according the current diagnosis criteria suggested in the protocol of the Ministry of Health.^(16)^ Of these, 28% (N=31) were not diagnosed during prenatal care (according to the criteria used in the Maternity during the period). This information may simplistically suggest that the previously used criteria may underdiagnose patients who nowadays would be considered diabetic, and who could evolve with complications associated with this pathology, such as macrosomia. Of the patients diagnosed with diabetes, the vast majority did not have satisfactory control, contributing to excessive fetal weight gain.

Still on maternal characteristics as possible risk factors for the occurrence of excessive fetal growth, a limitation recognized in the present study is the lack of data about the maternal pre-gestational Body Mass Index (BMI) and the patient’s weight gain during pregnancy. The clinical records analyzed did not have this information available for collection and these characteristics are described in the literature as important risk factors for excessive fetal growth, as was described in the scientific work published by K. Bowers et al.^(18)^ In view of this, it is suggested that health professionals involved in the patient’s hospital care pay attention to this data (which should theoretically be noted on the prenatal card), and which could provide valuable information for better assistance to the maternal-fetal binomial.

Regarding the reasons identified for hospitalization of patients who had macrosomic newborns, spontaneous labor was the main one. The second reason was advanced gestational age, identified in 24% of cases. The protocol for termination of pregnancy based on gestational age at the Maternity Hospital, during the period in which the work was carried out, provided for hospitalization and termination at 41 weeks of gestational age (if spontaneous labor did not occur, or reason for terminating the pregnancy before this gestational age). In the sample collected, most cases were full-term (355), but with more pregnancies close to being “post-term”, which influences birth weight. According to the NCHS, in 2014, the risk of birth weight greater than 4500 grams increases from 1.3% at 39-40 weeks to 2.9% at 41 weeks.^(19)^

Regarding the prevalence of mode of delivery in this study, there is a limitation in directly associating the chosen mode of delivery with the prenatal diagnosis of macrosomia. In other words, we cannot necessarily conclude that the predominant mode of delivery was cesarean simply because it was a sample of macrosomics. The indication for cesarean section may have been because the patient had a formal indication for terminating the pregnancy, such as, for example, a hypertensive disorder, but had a history of previous cesarean sections, which made it impossible, at the maternity’s discretion, to induce vaginal birth.

Of the 128 vaginal deliveries analyzed in the sample, 27 (21%) of them occurred with shoulder dystocia. The lack of consensus and objectivity in the criteria that define shoulder dystocia, which sometimes take clinical sense into account, sometimes are based on the use of obstetric maneuvers for its resolution, contributes to its incidence varying between 0.2 to 3 % of vaginal births.^(20)^ Studies involving the largest number of vaginal deliveries report incidences between 0.58% and 0.70%.^(21)^ Some authors relate its incidence to the weight of the newborn, ranging from 0.6 to 1.4% if it weighs between 2500 and 4000 grams and between 5% and 9% if the birth weight is between 4000 and 4500 grams.^(20)^ Additionally, in the presence of maternal diabetes, shoulder dystocia rates can reach 50%.^(22)^ The sample selected for this work consisted only of newborns weighing more than 4000 grams. Thus, despite the sufficient size, the sample becomes incomplete for certain comparative analyses, as these are newborns at the extreme right of the weight distribution curve. For example, there are researches showing an association between birth weight above 4500 grams and an increased incidence of shoulder dystocia. On the other hand, as we are already dealing with the sample of macrosomics only, this is not observed in our sample. Likewise, numerous other associations are not detected here, such as the correlation between macrosomia and birth trauma. With this, it was possible to demonstrate that some of the variables that are predictors of poor outcomes for the maternal-fetal binomial are not applicable when we already know that we are dealing with “extreme” cases. Such variables would be useful for classifying and predicting outcomes among the total number of deliveries.

However, it was still possible to identify some variables associated with poor outcomes, even among extreme cases. These are “Gestational age,” “Birth weight,” “Number of pregnancies,” number of deliveries (“Multiparous”) and “Birth weight > 4500 grams”. In fact, in the bivariate analysis, the variable “Diabetes mellitus” was also associated with jaundice in the newborn, as well as “Gestational age” but neither maintained statistical significance in the multivariate model. The multivariate model finds limitations in identifying which of these variables is more associated with the outcome (multicollinearity).^(14)^ One way to minimize this problem would be to collect a larger sample, which was not possible in this study.

Birth weight, as expected, continues to be relevant even among an extreme range of weights (above 4000 grams), and proved to be significant for the outcomes Hypoglycemia, Respiratory distress, APGAR score less than 7 in the 5th minute and of 3rd or 4th degree laceration.^(23)^

Regarding the occurrence of neonatal complications and hypoglycemia, both the weight directly and the categorization of weight between greater or less than 4500 grams were statistically significant. In the case of the 5th-minute APGAR score lower than 7, only birth weight above 4500 grams was associated. In the case of the occurrence of respiratory distress or severe lacerations, only birth weight in its quantitative form was associated, so that being greater, or less, than 4500 grams was indifferent.

However, in the case of 3rd and 4th degree lacerations, the direction of the association was the opposite of what was expected. The higher the weight of the newborn at birth, the lower the probability of the laceration. This result may be associated with variables not studied in this work. Such variables could relate to maternal anthropometric measurements, for example.

The lower the gestational age at birth, the greater the chances of neonatal complications, low APGAR in the 5th minute and ICU admission. Cases with some more severe outcomes had shorter gestation times, on average, indicating that a macrosomic newborn, whose weight is more associated with “near post-term” pregnancy, has fewer adverse outcomes than macrosomic deliveries. in “full-term” pregnancies.

Multiparous women were less likely to require an episiotomy. The fact of being multiparous was also statistically associated (negatively) with increased puerperal bleeding, and its explanation may lie in the type of delivery practiced.^(24)^

When is made a comparison between newborns with birth weight 4000-4500 grams and newborns with birth weight above 4500 grams (Table 4), a question remain unclear in the literature, we observed that only the outcomes “Neonatal Complications”, “Neonatal hypoglycemia” and “5-minute APGAR score of less than 7” had statistically significant results. Newborns with birth weight above 4500 grams has increased of risk of this outcomes.

The limitations of this review must be recognized, and further studies are needed to clarify what is the ideal cut-off point to define a “pathological macrosomia” and to propose an adequate mode of delivery for this group of newborns.

## Conclusion

The prevalence of macrosomia at the Maternity Hospital of the Hospital das Clínicas of UFMG from 2014 to 2019 was below the national average. Adequate identification of the maternal profile during prenatal care, with identification of risk factors for the occurrence of macrosomia, such as GDM, and its proper management, are essential to minimize complications. The birth weight of the newborn above 4000 grams was directly associated with the occurrence of neonatal complications in this study, such as hypoglycemia, respiratory distress, and 5th minute APGAR less than 7, especially if the weight was above 4500 grams. Gestational age was also associated with complications, the lower, the greater the risk. Shoulder dystocia was highly prevalent and, although a statistically significant association was not demonstrated in this study, the choice of mode of delivery must be judicious when there is suspicion of macrosomia.
